# The Tudor domain protein Tapas, a homolog of the vertebrate Tdrd7, functions in the piRNA pathway to regulate retrotransposons in germline of *Drosophila melanogaster*

**DOI:** 10.1186/s12915-014-0061-9

**Published:** 2014-10-06

**Authors:** Veena S Patil, Amit Anand, Alisha Chakrabarti, Toshie Kai

**Affiliations:** Temasek Life Sciences Laboratory, 1 Research Link, National University of Singapore, Singapore, The Republic of Singapore; Department of Biological Sciences, National University of Singapore, Singapore, The Republic of Singapore; School of Biological Sciences, Nanyang Technological University, Singapore, The Republic of Singapore; Current address: Department of Pediatrics, University of California, San Diego, School of Medicine, 9500 Gilman Drive, La Jolla, CA 92093 USA

**Keywords:** Germline, Nuage, piRNA, Tudor domain

## Abstract

**Background:**

Piwi-interacting RNAs (piRNAs) are a special class of small RNAs that provide defense against transposable elements in animal germline cells. In *Drosophila*, germline piRNAs are thought to be processed at a unique perinuclear structure, the nuage, that houses piRNA pathway proteins including the Piwi clade of Argonaute family proteins, along with several Tudor domain proteins, RNA helicases and nucleases. We previously demonstrated that Tudor domain protein Tejas (Tej), an ortholog of vertebrate Tdrd5, is an important component of the piRNA pathway.

**Results:**

In the current study, we identified the paralog of the *Drosophila tej* gene, *tapas* (*tap*), which is an ortholog of vertebrate *Tdrd7.* Like Tej, Tap is localized at the nuage. Alone, *tap* loss leads to a mild increase in transposon expression and decrease in piRNAs targeting transposons expressed in the germline. The *tap* gene genetically interacts with other piRNA pathway genes and we also show that Tap physically interacts with piRNA pathway components, such as Piwi family proteins Aubergine and Argonaute3 and the RNA helicases Spindle-E and Vasa. Together with *tej*, *tap* is required for survival of germline cells during early stages and for polarity formation. We further observed that loss of *tej* and *tap* together results in more severe defects in the piRNA pathway in germline cells compared to single mutants: the double-mutant ovaries exhibit mis-localization of piRNA pathway components and significantly greater reduction of piRNAs against transposons predominantly expressed in germline compared to single mutants. The single or double mutants did not have any reduction in piRNAs mapping to transposons predominantly expressed in gonadal somatic cells or those derived from unidirectional clusters such as *flamenco.* Consistently, the loss of both *tej* and *tap* function resulted in mis-localization of Piwi in germline cells, whereas Piwi remained localized to the nucleus in somatic cells.

**Conclusions:**

Our observations suggest that *tej* and *tap* work together for germline maintenance. *tej* and *tap* also function in a synergistic manner to maintain examined piRNA components at the perinuclear nuage and for piRNA production in *Drosophila* germline cells.

**Electronic supplementary material:**

The online version of this article (doi:10.1186/s12915-014-0061-9) contains supplementary material, which is available to authorized users.

## Background

Animal genomes have been invaded by a variety of transposons that propagate by populating the germline genome [1]. To combat the deleterious effects of invading transposons in the germline cells, host genomes have co-evolved an elegant RNA-based defense mechanism involving the Piwi-interacting RNAs (piRNAs) [2]. The piRNAs have been reported in many animals, such as *Drosophila*, rat, mouse and zebrafish [3-10]. In *Drosophila*, piRNA biogenesis involves two pathways: primary and secondary processing [9,10]. Primary processing, which involves Piwi, occurs in both somatic and germline cells of gonads. In this process, precursor transcripts from genomic clusters, which are specialized sites harboring fragmented transposons copies incapable of mobilization, are randomly processed into 23- to 29-nucleotide piRNAs that are in antisense orientation to the transposons. Secondary processing is a feed-forward loop that is also termed the ping-pong cycle [6,7]. The ping-pong cycle occurs only in germline cells and involves the two other Piwi family proteins, Aub and Ago3 (reviewed in [1]). This process is hypothesized to involve the cutting of transposon transcripts by Piwi/Aub-bound antisense piRNAs and the loading of resultant sense piRNAs onto Ago3. The Ago3 complex then cleaves antisense cluster transcripts for further processing into antisense piRNAs [6,7].

Aub and Ago3, along with many other proteins that are required for piRNA production in germline cells across species, localize to the nuage (‘cloud’ in French), a conserved perinuclear structure found in animal germline cells [2,11,12]. The evolutionarily conserved localization of piRNA pathway proteins makes the nuage a potential compartmentalized piRNA processing site in germline cells [2]. A majority of conserved nuage components contain Tudor domains, which bind symmetrically di-methylated arginine residues of Piwi family proteins and participate in the piRNA pathway [13-15]. We previously identified Tejas (Tej), a Tudor domain protein, as a germline piRNA pathway component [16]. The gene *tej* is required for transposon repression and localization of several piRNA pathway components to nuage, and Tej also physically interacts with the piRNA components Ago3, Aub, SpnE and Vas.

Here, we report the identification and characterization of *tapas* (which means ‘heat’ in Sanskrit, hereafter abbreviated as *tap*), a paralog of *Drosophila tej* and an ortholog of vertebrate *Tdrd7*. Tap is predominantly expressed in the germline cells and co-localizes with other piRNA pathway components. The gene *tap* genetically interacts with other piRNA pathway components, and Tap protein also physically interacts with the piRNA pathway components Ago3, Aub, SpnE and Va. Loss of *tap* leads to a milder derepression of a subset of retroelements that are repressed in the germline and a reduction in piRNAs mapping to them. However, when combined with the loss of *tej* function, the double mutants show loss of germline cells and a greater reduction in piRNA with more severe derepression of retrotransposons. Our results suggest that Tap functions synergistically with Tej in a complex to promote proper germline development and piRNA production.

## Results

### *tap* encodes a conserved Tudor domain protein that localizes to the nuage

**Figure 1:**
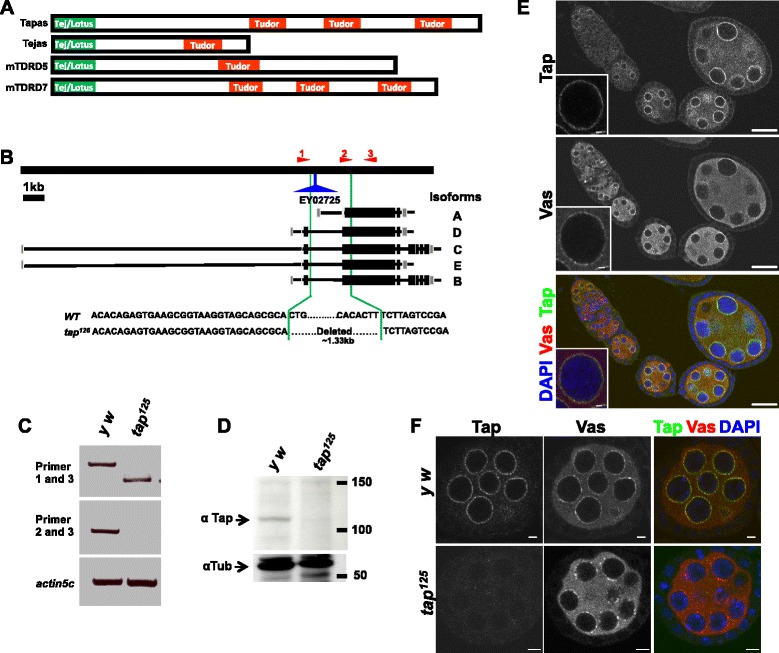
***tap*** **encodes a Tudor domain protein and localizes to the nuage. (A)** Schematic representation of the Tap and Tej proteins and their respective mouse orthologs, TDRD7 and TDRD5. Tap has three Tudor domains and a Tejas/Lotus domain in the C- and N-termini, respectively. **(B)** Schematic representation of the *tap* genomic locus. The *tap* gene is predicted to be transcribed in to five isoforms. The area between the green lines represents the deleted region in *tap* ^*125*^. **(C)** RT-PCR showing a truncated transcript in *tap* ^*125*^ ovaries. Primers 1, 2 and 3, which were used for RT-PCR, are indicated in (B) by the red arrowheads at the top. **(D)** Western blotting analysis using anti-Tap antibody detected a single band of approximately 110 KDa, which is closer to the predicted Tap size. The antibody could not detect a band at same position in *tap* ^*125*^-mutant ovaries. **(E,F)** Ovaries immunostained for Tap (green) and Vas (red). **(E)** Tap localizes to the perinuclear nuage with Vas. Scale bar =20 μm. Insets show a closer view of a single nurse cell nucleus. **(F)** Tap was undetectable in *tap* ^*125*^-mutant ovaries. Scale bar =5 μm.

We previously reported a Tudor domain protein Tej as a germline piRNA pathway component required for transposons repression and nuage localization of several other piRNA pathway components [16]. The *Drosophila* gene *CG8920* encodes its paralog, Tap. The orthologs of Tap and Tej, Tdrd7 and Tdrd5 respectively, are found in other animals, such as human, mouse, rat and zebrafish, and localize to the nuage [11,17-21]. Tap as well as Tdrd7 has three Tudor domains and a Tejas/Lotus domain (Figure 1A; [16,22,23]). Given its similarity with Tej, we addressed if Tap, like many other Tudor domain proteins, functions in the piRNA pathway in the germline [15,16,24-27].

To analyze *tap* function, we generated a deletion mutant through imprecise excision of a P-element, *EY02725*, inserted within an intron of the *tap* gene. The resulting allele, *tap*^*125*^, lost a 1.33-kb genomic region encompassing a portion of the longest common exon shared by all putative *tap* isoforms (Figure 1B). RT-PCR confirmed a truncation of the *tap* transcript in *tap*^*125*^-mutant ovaries (Figure 1C). Western blotting with anti-Tap antibody detected a band corresponding to the predicted size of Tap protein, which was absent in *tap*^*125*^-mutant ovaries (Figure 1D), indicating *tap*^*125*^ is a loss-of-function allele. Similar to its paralog Tej, Tap expression was observed only in germline cells and localized to the perinuclear foci in all germline cells except oocytes (Figure 1E,F; [16]). Immunostaining showed that most of the Tap foci co-localized with well-known nuage components, Vas and Tej (Figure 1E,F; Additional file 1: Figure S1A; [16,28]), though there were few distinct foci of each of those, suggesting that Tap is a nuage component. The Myc-tagged Tap protein expressed from a transgene also co-localized with Vas at the perinuclear nuage when expressed by the germline driver nanosGAL4 (Additional file 1: Figure S1B). Unlike Vas, however, endogenous Tap and Myc-Tap localized only to the nuage and not to the pole plasm (Additional file 1: Figure S1C; [29,30]). The perinuclear localization of Tap was undetectable in *tap*^*125*^ ovaries (Figure 1F), which confirms the specificity of the antibody and the perinuclear localization of Tap. Nuage localization of Tap was further confirmed by examining a protein trap line, *CC00825*, expressing GFP-Tap (Additional file 1: Figure S1D; [31]).

### *tap* likely shares a synergistic functional relationship with its ortholog *tej* for the germline development

**Figure 2:**
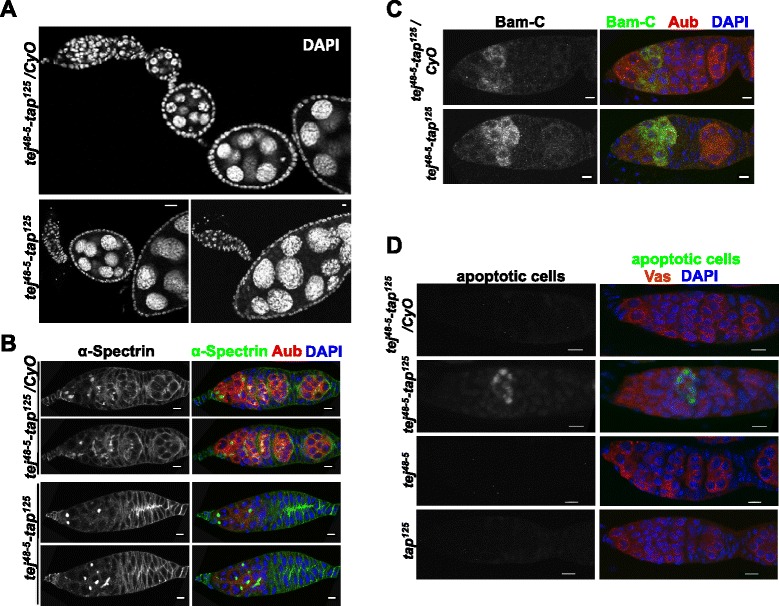
**The** ***tej*** ^***48–5***^ ***-tap*** ^***125***^ **double mutant exhibits more severe defects during oogenesis. (A)** DAPI staining of *tej* ^*48–5*^ *-tap* ^*125*^ double-mutant ovarioles shows defects in the germline development. **(B)** Immunostaining with α-Spectrin (green) and Aub (red), and DNA staining with DAPI (blue) show intact germline stem cells in *tej* ^*48–5*^ *-tap* ^*125*^ mutants and heterozygous ovaries but cysts appeared to be arrested in mostly the two- to four-cell stage in *tej* ^*48–5*^ *-tap* ^*125*^ double-mutant ovaries (bottom two panels). Scale bar = 5 μm. **(C)** Immunostaining for Bam (green), Aub (red) and DAPI (blue) shows a comparable Bam expression pattern between *tej* ^*48–5*^ *-tap* ^*125*^ mutant and heterozygous controls. Scale bar = 5 μm. **(D)** Terminal deoxynucleotidyl transferase dUTP nick-end labeling staining shows dying germline cells in *tej* ^*48–5*^ *-tap* ^*125*^ double-mutant germarium, but not in heterozygous control ovarioles or either single mutant. Scale bar = 5 μm.

The female and male homozygotes of *tap*^*125*^ as well as trans-heterozygotes, *tap*^*125*^ over *Df(2R)BSC19* uncovering the *tap* locus, were viable and fertile, indicating that *tap* function is dispensable for *Drosophila* viability and fertility under laboratory conditions. Although Tap localized with Vas at nuage, *tap*^*125*^ did not display any of the severe phenotypes observed in other mutants of piRNA pathway components that localize to the nuage, such as sterility, defective karyosome formation, DNA double-strand breaks and failure in polarity formation (reviewed in [2]). The similarity in *tej* and *tap* gene structures prompted us to examine the possibility that these two genes have a functional relationship. To assess this possibility, we generated a *tej-tap* double mutant by recombining *tej*^*48-5*^and *tap*^*125*^alleles. The double-mutant females were sterile and males were fertile only for the first few days after eclosion, whereas both *tej*^*125*^ and *tap*^*48–5*^ single-mutant males were fertile [16]. The *tej*^*125*^-*tap*^*48–5*^ double-mutant gonads showed severe degeneration: by seven days post eclosion (dpe) most of the double-mutant ovaries became atrophic and were very small compared to the heterozygote control, whereas both *tej*^*48–5*^ and *tap*^*125*^ single-mutant ovaries were visibly similar in size compared to the controls (Additional file 1: Figure S2A). The degeneration of double-mutant ovaries usually started after three dpe; most of the four to six dpe ovarioles showed germaria containing very few germline cells attached to very late stage egg chambers (Figure 2A). A very small percentage of two-dpe double-mutant ovaries showed this phenotype. A similar degeneration of germline cells was also observed in the male gonads (Additional file 1: Figure S2C). Hence in subsequent experiments, unless otherwise noted, we analyzed one- to two-dpe double-mutant flies to avoid any defects that may have been caused by gonad degeneration. Our data suggest that the double mutants may have defects in the maintenance and/or division and differentiation of germline stem cells (GSCs).

To study the maintenance of GSCs and their differentiation, we examined the fusomes and *bag-of-marbles* (*bam*) expression by immunostaining (Figure 2B,C and Additional file 1: Figure S2B; [32,33]). We observed unbranched round fusomes in germline cells at the tip of female germaria and around the hub in testes in both control and double-mutant gonads that started losing germline cells, suggesting GSCs are maintained in double-mutant gonads. However, while 2-, 4-, 8- and 16-cell cysts were discernible by branched fusomes in the controls, most of the cysts in *tej*^*48–5*^*-tap*^*125*^ double mutants appear to be arrested at the two-cell stage, though some four- or eight-cell cysts were also observed (Figure 2B and Additional file 1: Figure S2B). Differentiation factor Bam expression pattern in double-mutant ovaries was similar to that in controls, suggesting no defects in differentiation (Figure 2C). To investigate the cause of loss of germline cells, we performed a terminal deoxynucleotidyl transferase-mediated dUTP nick-end labeling (TUNEL) assay (Figure 2D and Additional file 1: Figure S2C). We observed apoptotic cells in the germarium and in the testis of the double mutants, while no TUNEL-positive germline cells were discernible in the control or either single-mutant germaria or the control testes. Approximately 50% (n =30) germaria had TUNEL-positive cells in two-dpe *tej*^*48–5*^*-tap*^*125*^ double*-*mutant ovaries: they start appearing in region 2 in germarium. The apoptosis in germline cells likely explains loss of germline cells in region 2b and late stage egg chambers attached to germarium of four- to seven-dpe *tej*^*48–5*^*-tap*^*125*^ flies (Figure 2A,B).

To examine any defect in the polarity determination, we stained ovaries with dorsal marker Gurken. While *tap*^*125*^-mutant oocytes showed normal anterior-dorsal Gurken localization as we previously observed in *tej* mutants, Gurken expression was not detectable in the *tej*^*48–5*^*-tap*^*125*^ double-mutant ovaries, indicating a severe defect in polarity establishment (Additional file 1: Figure S2D; [16]). Consistent with this observation, the eggs laid by the double-mutant females were also devoid of dorsal appendages (data not shown). Taken together, these results suggest that *tap* and *tej* are required together for proper germline survival, development and polarity formation in gonads.

### *tap* is dispensable for fertility but is required for retroelement repression

**Figure 3:**
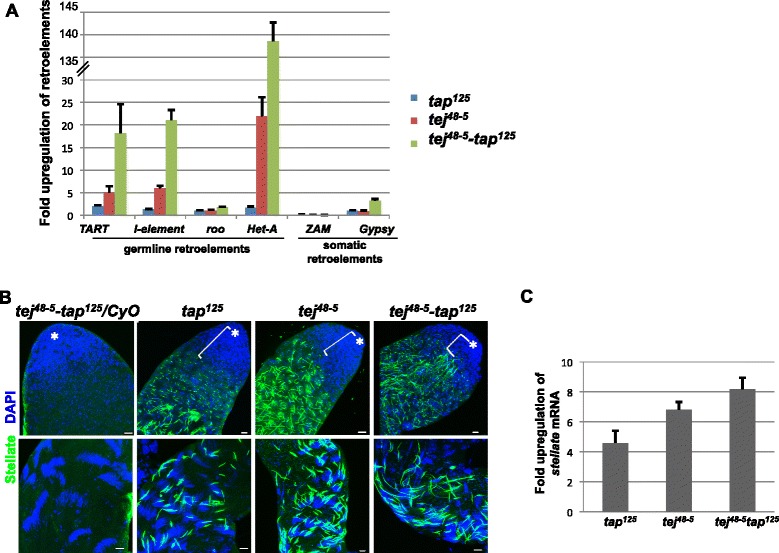
***tap*** **is required for retroelement repression and likely functions with** ***tej*** **. (A)** Quantitative RT-PCR (qRT-PCR) showing fold increase in expression of few representative retroelements in *tej* ^*48–5*^ *, tap* ^*125*^ and *tej* ^*48–5*^ *-tap* ^*125*^ mutant ovaries compared to respective heterozygous controls. The *tap* ^*125*^-mutant displays milder upregulation of *TART*, *I-element*, *roo* and *Het-A*, whereas the *tej* ^*48–5*^ *-tap* ^*125*^ double mutant exhibits much more severe upregulation of these retroelements compared with both *tej* ^*48-5*^and *tap* ^*125*^ single mutants. Error bars represent standard deviations. **(B)** *tap-*, *tej-* and the double-mutant testes stained for Ste (green) and DAPI (blue). Top and bottom panels show the apex and posterior regions, respectively. Asterisks mark the hub of the testis. Square brackets denote the distance from the hub to the point where Ste crystals start to appear. Scale bar = 20 μm. **(C)** qRT-PCR showing increase in *ste* transcript levels in the *tap* ^*125*^-mutant testes compared to their respective heterozygote controls, and confirms more severe Ste crystal formation in *tej* ^*48–5*^ *-tap* ^*125*^ double mutants. The error bars represent the standard deviation.

**Figure 4:**
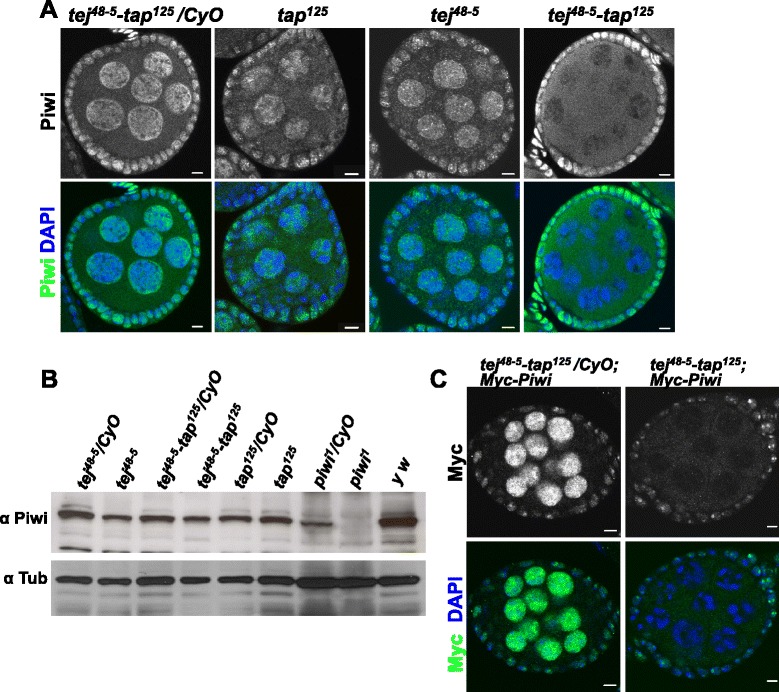
**Piwi fails to localize to the nucleus in germline cells of the** ***tej-tap*** **double mutant. (A)** Ovaries stained for Piwi (green) and DAPI (blue). Piwi localized to the nucleus in the somatic cells but failed to localize to the nucleus of germline cells of the *tej* ^*48–5*^ *-tap* ^*125*^ double mutants. **(B)** Western blotting with anti-Piwi antibody showing no significant difference in Piwi expression levels in *tej* ^*48–5*^ *-*, *tap* ^*125*^ *-* and *tej* ^*48–5*^ *-tap* ^*125*^-mutant ovaries in comparison to their respective heterozygous controls. The same blot was re-probed with anti-α-Tubulin to examine loading. **(C)** Expression of Myc-Piwi in *tej* ^*48–5*^ *-tap* ^*125*^ ovaries does not rescue nuclear localization of Piwi in germline cells. Immunostaining shows cytoplasmic Myc-Piwi in germline cells of *tej* ^*48–5*^ *-tap* ^*125*^ double-mutant ovaries. Scale bar = 5 μm.

Loss of function of many nuage components leads to derepression of retroelements in animal gonads (reviewed in [1,2]). We examined whether *tap* also participates in retrotransposon repression by comparing expression levels of representative retroelements between *tap*^*125*^ mutants and heterozygous control ovaries with quantitative RT-PCR (qRT-PCR). The *tap*^*125*^-mutant ovaries exhibited a slight upregulation of *TART*, *HeT-A* and *I-element*, which are targeted by piRNAs in germline cells, but there was no significant effect on the expression of *ZAM* and *Gypsy*, which are regulated by piRNAs in gonadal somatic cells (Figure 3A). The *tap*^*125*^-mutant testes also displayed high expression of the Stellate (Ste) protein, which is repressed by *su(ste)* piRNA (Figure 3B; [4]). qRT-PCR also revealed significant upregulation of *ste* transcript in *tap*^*125*^-mutant testes compared to heterozygote controls (Figure 3C). These results suggest that *tap* function, although dispensable for fertility in *Drosophila*, is required for transposon repression in germline cells.

The *tej*^*48–5*^*-tap*^*125*^ double-mutant females exhibited more severe derepression of reteroelements targeted by piRNAs in germline cells compared to either single mutant (Figure 3A; [10]). By contrast, expression levels of reteroelements, which are predominantly expressed in somatic cells, did not differ significantly in the double mutants compared with the heterozygous controls (Figure 3A). Similarly, expression of Ste protein and transcript in the male germline was higher in the double mutants than in either single mutant (Figure 3B,C). Ste crystals were longer and appeared earlier during gametogenesis in double mutants (square brackets in Figure 3B). These results suggest that *tej* and *tap* are functionally related for transposon repression in the *Drosophila* germline.

### *tap* likely functions synergistically with *tej* for localization of piRNA pathway components in the germline

Many nuage and/or piRNA components genetically interact in *Drosophila* as well as in other systems such as mouse: they appear to be interdependent for their proper localization to the nuage [9,16,21,24,26,34-38]. We examined whether *tap* function is also required for localizing other nuage components to the nuage and vice versa. Although all of the examined nuage components - Vas, Qin, Tej, Aub, Ago3, Krimp and Mael - localized to the perinuclear nuage, they often formed larger aggregates in *tap*^*125*^-mutant ovaries (Additional file 1: Figure S3A). In reciprocal experiments, in which we examined Tap localization in nuage component mutants, Tap remained unaffected and formed perinuclear foci in all the examined mutants (Additional file 1: Figure S3B). These results suggest that *tap* alone likely has a minor role in localization of examined nuage components. We previously showed that Vas, but not the other piRNA components such as Aub, Ago3, Krimp and Mael, depends on *tej* function [16]. By contrast, in the absence of both *tej* and *tap* function, Vas, as well as all examined piRNA pathway components, was displaced to cytoplasm from the perinuclear nuage (Additional file 1: Figure S3C).

Defects in localization of piRNA component proteins in *tej*^*48–5*^*-tap*^*125*^ ovaries indicates that *tej* and *tap* likely function together for their localization. To address the nature of this functional relation between *tej* and *tap*, we performed complementation analysis (Additional file 1: Figure S4). Tap expression either in *tej*^*48–5*^*-tap*^*125*^ double-mutant or in *tej*^*48–5*^ single-mutant germline cells failed to rescue the defects in perinuclear localization of Krimp, Aub and Ago3. However, Tej expression in double mutants brought them back to the perinuclear region, although they formed larger foci than the heterozygous controls, which was reminiscent of their localization in *tap*^*125*^ mutants (Additional file 1: Figure S3A). In summary, overexpression of either Tej or Tap in *tej*^*48–5*^*-tap*^*125*^ mutants manifests the observed phenotypes in the reciprocal single mutants, suggesting that Tej and Tap cannot complement each other’s function and likely have a synergistic relationship for localization of piRNA pathway components.

### *tej* and *tap* together are required for nuclear localization of Piwi in germline cells

The other distinct defect occurring in the *tej*^*48–5*^*-tap*^*125*^ double mutant but not in either the *tej*^*48–5*^ or *tap*^*125*^ mutants was the cytoplasmic localization of Piwi in germline cells, whereas in the heterozygous controls Piwi localized to the nucleus (Figure 4A and Additional file 1: Figure S5A). In the gonadal somatic cells, however, Piwi remained localized to the nucleus in the double mutants, like those in the heterozygous controls. The Piwi expression levels in *tej*^*48–5*^*-tap*^*125*^*, tej*^*48–5*^ and *tap*^*125*^ mutants were found to be comparable to those in respective heterozygotes by western blot analysis (Figure 4B), suggesting that loss of *tej-tap* function together leads to Piwi mis-localization without affecting its expression level. Furthermore, Myc-Piwi expressed from a native promoter in addition to endogenous Piwi in *tej*^*48–5*^*-tap*^*125*^ double-mutant germline cells remained in the cytoplasm, whereas it localized to the nucleus in the somatic follicle cells (Figure 4C and Additional file 1: Figure S5B), suggesting that simultaneous impairment of *tej* and *tap* functions prevents Piwi from entering the nucleus in germline cells. In addition, the localization pattern of another primary piRNA pathway component, Armitage, in the double-mutant gonadal somatic cells remained comparable to that in control (Additional file 1: Figure S5C; [39]). These results suggest that loss of *tej* and *tap* together does not affect the localization of piRNA pathway components in gonadal somatic cells.

### Tap physically interacts with piRNA pathway components

**Figure 5:**
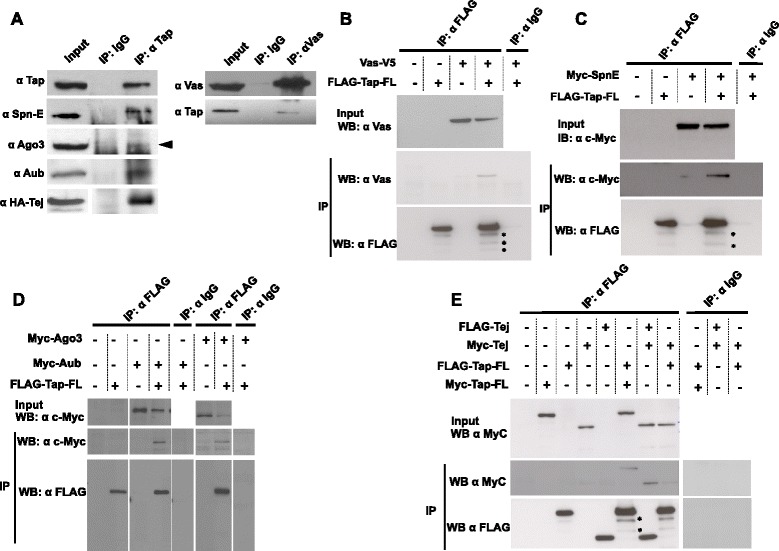
**Tap physically interacts with other components of the ping-pong amplification pathway. (A)** Immunoprecipitation with anti-Tap antibody using ovarian lysates followed by western blotting. Vas, Aub, Spn-E, Tej and a trace amount of Ago3 (arrowhead) were pulled down along with Tap. **(B-E)** S2 cells were co-transfected with FLAG an octa-peptide (DYKDDDDK)-Tap along with V5-Vas, Myc-Spn-E, Myc-Aub, Myc-Ago3, FLAG-Tej and Myc-Tap, and the cell lysates were subjected to immunoprecipitation with anti-FLAG antibodies. IgG was used as control in all IP experiments. **(B,C)** V5-Vas and Myc-Spn-E were pulled down with FLAG-Tap but not with IgG. **(D)** Both Aub and Ago3 were pulled down with FLAG-Tap but not with the control IgG. **(E)** FLAG-Tap was pulled down and blotted with FLAG or Myc antibody. A small amount of Tap precipitated with Tej as well as with itself. Similarly, Myc-Tej was also pulled down with FLAG-Tej. IgG: immunoglobulin G, IP: immunoprecipitation, WB: western blotting.

Tej was previously shown to physically interact with some of the piRNA pathway proteins such as Vas, Spn-E and Aub [16]. To examine if Tap also physically interacts with some piRNA pathway components, immunoprecipitated Tap from ovarian lysate with the anti-Tap antibody was examined for interaction with some piRNA pathway components (Figure 5A). Indeed, Spn-E, Aub and Tej were pulled down along with Tap, suggesting that Tap interacts with those in the germline. However, we detected a very small amount of Ago3 in Tap immunoprecipitate, suggesting a weak interaction between Tap and Ago3. We also observed Tap and Vas interaction in a reciprocal manner where we detected Tap in anti-Vas immunoprecipitate.

We further confirmed these interactions in S2 cells; FLAG (DYKDDDK)-tagged Tap was transfected separately with V5-tagged Vas, Myc-tagged Spn-E, Myc-tagged Aub, or Myc-tagged Ago3 (Figure 5B-D). We pulled down FLAG-Tap and detected tagged Vas, Aub, Ago3 and Spn-E in immunoprecipitate, suggesting that Tap interacts with them in the absence of any other germline factors. To confirm interaction between Tej and Tap, we co-transfected Myc- and FLAG- tagged Tap and Tej in S2 cells and performed immunoprecipitation (IP). Tap and Tej co-immunoprecipitated with each other in reciprocal IP experiments, suggesting that they interact in the absence of any other germline proteins (Figure 5E). Taken together, our IP experiments suggest that Tap can interact not only with its paralog, Tej, but also with other piRNA pathway components. The observed physical interaction of Tap with Tej and other piRNA pathway components supports our earlier observation of functional relationships between Tap and Tej for germline development, piRNA production and transposon repression.

### The *tap-*, *tej-* and *tap-tej* mutants display defects in piRNA production in germline

**Figure 6:**
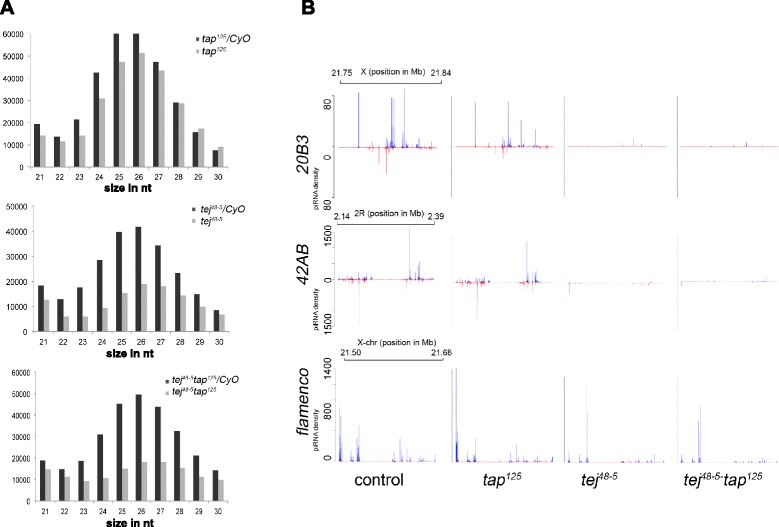
**The** ***tap*** **gene is required for robust piRNA production against germline transposable elements, while** ***tap*** **along with** ***tej*** **is required to maintain piRNA population in germline. (A)** Abundance of genome-matching unique reads, 21 to 29 nucleotides in length, in *tap* ^*125*^ *-*, *tej* ^*48–5*^ *-* and *tej* ^*48–5*^ *-tap* ^*125*^-mutant ovaries and their respective heterozygous controls. The *tap* mutants showed reduced numbers of 23- to 29-nucloetide small RNAs, but the *tej* ^*48–5*^ *-tap* ^*125*^ double mutants showed the most severe reduction in comparison to both single mutants. **(B)** Mapping of the 23- to 29-nucloetide sequences to bidirectional piRNA clusters at *20B3* and *42AB*, and a single-strand piRNA cluster, *flamenco*. A feeble reduction in bidirectional cluster mapping piRNAs in *tap* ^*125*^ mutants was discernible compared to *tej* ^*48–5*^ mutants, while *tej* ^*48–5*^ *-tap* ^*125*^ double mutants had a greater reduction than both single mutants.

Next, to understand the roles of *tap* and *tej* in piRNA biogenesis, we performed deep sequencing of small RNAs isolated from *tej*^*48–5*^, *tap*^*125*^, *tej*^*48–5*^*-tap*^*125*^ and their respective heterozygous ovaries. The small RNA libraries were aligned to the genome and canonical transposons, and then normalized with small nucleolar RNA-derived small RNAs, genic-endo-siRNAs and non-coding RNAs with respective heterozygous controls (Additional file 2: Table S1). We examined small RNAs, from 23 to 29 nucleotides in size, for subsequent piRNA analysis, and considered antisense piRNAs to compare transposon-mapping piRNA levels between mutants and controls. The *tap*^*125*^-mutant ovaries only had a 16% reduction in the number of genome-matching unique reads compared with the heterozygous control (Figure 6A). *tap*^*125*^ mutants did not show any significant decrease in the overall cluster-mapping piRNAs, including those mapping to the largest bidirectional cluster at *42AB*. However, some other bidirectional clusters, such as those at *20B3*, *62A*, *80E*, *3LHet* and *3RHet* in *tap*^*125*^ mutants, had a significant reduction in piRNAs (25% to 50%) (Figure 6B; Additional file 3: Table S2). These clusters housed several transposable elements (TEs) including *TART-A*, *I-element*, *Het-A* and *roo,* which showed slight depression in *tap*^*125*^-mutant ovaries (Figure 3A), suggesting that the reduction in piRNAs may have caused the observed derepression.

**Figure 7:**
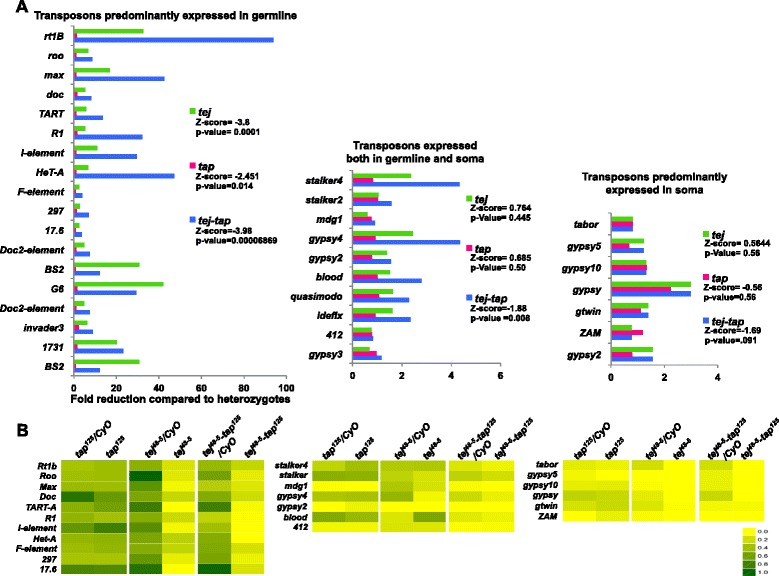
***tej*** **and** ***tap*** **likely function together for production of cluster-derived and ping-pong piRNAs. (A)** Bar plots showing reduction separately for piRNAs mapping to transposons families expressed predominantly in germline, both germline and soma, and predominantly in soma. The *tej* ^*48–5*^ *-tap* ^*125*^ mutants showed a more severe reduction in piRNAs targeting transposons expressed in germline and both germline and soma compared to that in both single mutants. **(B)** Heat maps representing the ratio of ping-pong piRNAs (10-nucleotide overlap) to piRNA pairs with overlaps of 2 to 26 nucleotides mapping to transposable elements (TEs) of three different subsets: those predominantly expressed in the germline (top panel), in both the germline and soma (middle panel), and predominantly in somatic cells (bottom panel). *tap* ^*125*^ mutants had a less severe reduction in the ping-pong piRNAs mapping to TEs predominantly repressed in germline than the *tej* ^*48–5*^ *-tap* ^*125*^ mutants, which suffered the most severe loss in ping-pong ratios compared to both single mutants.

The overall transposon-mapping piRNAs were also not significantly reduced in the *tap* mutants. However, we observed up to 57% reduction in the piRNAs mapping to a subset of transposons known to be repressed by piRNAs in germline, such as *doc*, *Rt1b*, *TART-B*, *I-element* and *roo,* in comparison to heterozygous controls (Wilcoxon *t*-test; Z-score -2.45, *P* = 0.01; Figure 7A; Additional file 4: Table S3; [10]). We also observed a 10% to 49% reduction in ping-pong piRNAs mapping to these transposons in *tap* mutants (Figure 7B). By contrast, *tap*^*125*^ mutation did not lead to any statistically significant decrease in the piRNAs mapping to transposons targeted by piRNAs in both germline and somatic cells and those predominantly in in somatic cells. This indicates that *tap* is likely required for production of piRNAs for a subset of transposons in germline cells.

Though the loss of *tap* homologue, *tej*, itself led to a significant decrease in the overall piRNA levels, *tej*^*48–5*^*-tap*^*125*^ double mutants exhibited further reduction in piRNAs (Figures 6 and 7), suggesting that *tap* also functions with *tej* for piRNA production. The overall genome-mapping piRNAs were more severely reduced in the *tej*^*48–5*^*-tap*^*125*^ double mutants (64%) than in the *tej*^*48–5*^ mutants (53%) (Figure 6A). Similarly, loss of both *tej* and *tap* led to a greater reduction in cluster mapping piRNAs (82% reduction in comparison to the heterozygous control) than *tej* mutant alone (74% reduction in comparison to heterozygous control; Additional file 1: Figure S6A, Additional file 3: Table S2). The *tej*^*48–5*^*-tap*^*125*^ double-mutant ovaries had fewer piRNAs mapping to all bidirectional clusters, including that at *42AB*, compared to single mutants (Figure 6B and Additional file 3: Table S2). The double mutant also showed a greater reduction in the proportion of cluster mapping reads with U at the first position compared with both single mutants (Additional file 1: Figure S6A,B). These results suggest that loss of *tej* and *tap* together causes a more severe reduction in cluster-derived piRNA production in germline cells. We did not observe a significant reduction in piRNAs derived from *flamenco* or other major unidirectional clusters in single or double mutants (Figure 7A and Additional file 3: Table S2), further supporting a germline-specific function of *tej* and *tap* in piRNA production. The slight decrease (10%) in flamenco-mapping piRNAs in the double mutants could be a secondary effect resulting from the distortion of ovarian structure in the *tej*^*48–5*^*-tap*^*125*^ mutants.

The *tej*^*48–5*^*-tap*^*125*^ mutants also showed a more severe reduction in overall transposon-mapping piRNAs (73% overall and 81% in antisense piRNAs), compared with *tej* mutants (62% overall and 70% in antisense) (Additional file 1: Figure S6C,D; Additional file 4: Table S3). The piRNAs of 26 to 29 nucleotides in length were more severely reduced in the *tej*^*48–5*^*-tap*^*125*^ mutants than in the *tej*^*48–5*^ mutants (Additional file 1: Figure S6D).

The piRNAs mapping to transposons predominantly targeted in germline were more severely reduced in the *tej*^*48–5*^*-tap*^*125*^ mutants (88%, Z-score: −3.98, *P = *0.00006,) than the *tej* mutants (80%, Z-score: −3.8, *P = *0.0001; Figure 7A, left panel). Similarly, only the *tej*^*48–5*^*-tap*^*125*^ double-mutant ovaries had a significant reduction in piRNAs mapping to the transposons targeted by both germline and somatic piRNAs (29%, Z-score −1.88, *P = *0.008) while no significant change in these piRNAs was observed in *tej*^*48–5*^ mutants (10%, Z-score: 0.746, *P = *0.445; Figure 7A, middle panel). However, no significant loss in piRNAs targeting transposons predominantly expressed in somatic cells was observed in *tej*^*48–5*^*-tap*^*125*^ double mutants or *tej*^*48–5*^ mutants (Figure 7A, right panel). Notably, in *tej* single mutants, piRNAs mapping to *blood*, *HMS-Beagle* and *rover* were increased compared with the heterozygous control (Additional file 4: Table S3); while the antisense piRNAs matching to these transposons were reduced, sense piRNAs were largely increased. However, no such increase in sense piRNAs was observed in *tej*^*48–5*^*-tap*^*125*^ double mutants (Additional file 5: Table S4).

The double mutants also had an overall higher reduction in the transposon-mapping sense piRNAs with A at 10th position and antisense piRNAs with U at 1st position (Additional file 1: Figure S6E), indicating a defect in the secondary processing. To analyze this, we calculated the ratio of sense-antisense piRNA pairs with a 10-nucleotide overlap to piRNA pairs with any overlap in length. The *tej*^*48–5*^*-tap*^*125*^ double mutants showed a more severe decrease in the above ratios for transposons expressed in the germline and in both germline and soma than the *tej* single mutants. This was also supported by greater loss in the ping-pong piRNA pairs in double mutants than in the *tej* mutants (96% versus 90%, compared with those in each heterozygous control, respectively; Additional file 1: Figure S6E). These observations are consistent with a greater derepression of transposons in *tej*^*48–5*^*-tap*^*125*^ double mutants than that in *tej* single-mutant ovaries (Figure 3). More severe loss in ping-pong piRNAs in double mutants also emphasizes the requirement of *tej* and *tap* together for secondary piRNA production, and supports the observed synergistic relationship between them for piRNA production.

## Discussion

In this study we have characterized Tap, the paralog of Tej, which was previously reported as an essential piRNA pathway component that localizes to the nuage [16]. Like Tej, Tap is expressed predominantly in germline cells. *tap* genetically and physically interacts with Piwi family proteins and other piRNA pathway components. A reduction in germline piRNAs and upregulation of TE and *ste* expressions confirm participation of *tap* in piRNA pathways. Similarly, mouse ortholog of Tap, Tdrd7, was reported to be involved in the suppression of the retrotransposon *LINE1* and localizes to chromatoid bodies, which is the equivalent structure of the *Drosophila* nuage [17,21]. In Tdrd7-knockout mouse testes, however, piRNA production is not affected, but *LINE1* appears to be translationally upregulated [21]. This could be because Tdrd5 may have a more robust role in piRNA production in vertebrates than that in *Drosophila*, and the loss of Tdrd7 may be fully compensated for by Tdrd5. Analysis of piRNAs in Tdrd5 and Tdrd7 double-knockout mouse would shed light on their potential synergistic function in vertebrate. In addition, unlike Tap, mouse Tdrd7 is also expressed in somatic tissue - lens fiber cells - and its loss of function results in defects in spermatogenesis and somatic phenotypes such as cataract and glaucoma in mouse and human [21,40]. This suggests that, while mammalian Tdrd7 has a wider role in gonads and somatic tissues, Tap may have a specific role in *Drosophila* gonads. However, we cannot eliminate the possibility that *tap* is expressed at a very low level in somatic tissues and that its function is dispensable in the laboratory environment.

The absence of robust phenotypes in *tap*^*125*^ mutants questions the importance of *tap*. By contrast, loss of function of its paralog *tej* causes severe reduction in piRNAs in germline and mis-localization of several piRNA proteins from the nuage (Figures 6 and 7; Additional file 1: Figure S3; [16]). Our study with the *tej*^*48–5*^*-tap*^*125*^ mutants underscored the importance of the synergistic function of *tap* and *tej*. Loss of *tej* and *tap* together leads to the loss of germline cells by apoptosis in the germarium (Figure 2D), indicating that they function together to maintain early germline cells. In late stages, *tej* and *tap* together are required for polarity formation, which is indicated by loss of Gurken expression (Additional file 1: Figure S2D). In addition, we observed mis-localization of all examined nuage components and a statistically more robust reduction in cluster- and transposon-mapping piRNAs in double mutants (Figures 6 and 7; Additional file 3: Table S2 and Additional file 4: Table S3). These results also indicate that Tej and Tap function together for the piRNA pathway, and explain the higher derepression of TEs in double mutants than in *tej* mutants. The reduction in the ratio of antisense piRNAs having U at the first position and in ping-pong-derived piRNAs was also more severe in double mutants than in either single mutant. These defects suggest that Tej and Tap together could support primary processing, probably by engaging Aub and/or Piwi, and a more robust ping-pong amplification cycle for piRNA amplification.

A synergistic relationship between *tej* and *tap* for piRNA production is also suggested by the mis-localization of Piwi from the nucleus in germline cells of double mutants, while Piwi stayed in the nucleus of germline cells in both single mutants (Figure 4 and Additional file 1: Figure S5A). A recent study showed that the ablation of piRNA binding ability of Piwi leads to its retention in the cytoplasm of germline cells [41]. Hence, it may also be possible that Tej and Tap together are required for the piRNA loading onto Piwi in the germline cells of *Drosophila*. Overexpression of either *tej* or *tap* in the double mutant did not fully rescue the phenotype of the double mutants (Additional file 1: Figure S4), suggesting that they are not functionally redundant, but act synergistically for piRNA pathway and germline development. Similarly, Tdrd7 and Tdrd6 double-knockout mice exhibited more severe defects in chromatoid body and Miwi localization than single mutants [21,42], suggesting a conservation of functional relationship among piRNA components across species. However, it is currently unclear whether the more severe defects in germline development in the double mutants are correlated with heavy loss of piRNAs or if *tej* and *tap* have any piRNA-independent role in germline development.

## Conclusions

We here report on *tap*, a paralog of a germline piRNA pathway protein *tej.* Although the *tap* functions in the piRNA pathway, a milder derepression of transposons and milder decrease in piRNAs indicate it probably does not have a robust role in the piRNA pathway. However, *tap* likely functions together with *tej* for maintenance of germline cells in early stages and proper development of the germline, which is reflected by apoptosis in germline cells, atrophic ovaries, and loss of Gurken expression in double-mutant ovaries. We also showed that *tej* and *tap* function in a synergistic manner in a complex for piRNA production to safeguard the germline genome from transposons. Our findings describe a functional relationship between two germline piRNA pathway components. We believe that studies on functional relationships between piRNA pathway components might prove helpful in elucidating the mechanistic understanding of the piRNA pathway.

## Methods

### *Drosophila* strains

Either *y w* or the respective heterozygote was used as a control. A loss-of function allele of *tap*, *tap*^*125*^ was isolated from more than 150 independent excision lines of a P-element insertion line, *P{EPgy2}G8920[EY02725]/CyO*, by PCR-based screening using the primers Tap 1Fw (AGCCTTTTACTCCTTTGGAACC) and Tap 2 Rv (CGACTTCCTTCGTTATTTGACC). The *tap*^*125*^ allele lost approximately 1.33 kb in the locus and instead contained a 28-nucleotide insertion, which is possibly a remnant of the P-element. The mutant alleles and allelic combinations used in the study were *tap*^*125*^, *tap*^*125*^*/Df(2R)BSC19*, *tej*^*48–5*^ [16], UASp-Venus-Tej (in this study), UASp-Myc-Tap (in this study), *qin*^*M41–13*^ (previously designated *kumo*^*M41–13*^; [26]), *mael*^*M391*^*/Df(3 L)79E-F* [43,44], *vas*^*PH165*^ [45], *spn-E*^*616/hls3987*^ [46,47], *aub*^*NH2/N11*^ [48,49], *ago3*^*t2/t3*^ [9], *krimp*^*f06583*^ [24], *piwi*^*1*^ [50], Myc-Piwi [51] and the *tap* protein trap line *CC00825* [31]. The *tej*^*48–5*^-*tap*^*125*^double mutant was generated by recombination and was screened by a PCR-based method.

The full-length Tap coding sequence was amplified by PCR with the primers CACC-ATGGAAAAGCAGGAGGTC and TGTTGCTGGCTGTGCGTGCTT, using the cDNA generated from ovarian RNA and cloned into pENTR™/D-TOPO (Invitrogen, life technologies Grand Island, NY, USA) in accordance with the manufacturer’s protocol. The resulting pENTR Tap and previously generated pENTR Tej were recombined into pPMW and pPVW, respectively [16]. The pPMW-Tap and pPVW-Tej plasmids were injected into *y w* embryos to generate transgenic flies using a standard protocol [52]. The expression of transgenes was driven in the germline by *nosGal4VP16* [53].

### Antibody generation

Rat anti-Tap antiserum was generated against a portion of His-tagged Tap (amino acids 26 to 159). The corresponding fragment was amplified with the primers Tap antigen Fw (CACC-ACGCTGCGGTCCATCGTC) and Tap Antigen Rv (TTA-GCCCGTTAGATCTTGTTT). After cloning into pENTR™/D-TOPO, this sequence was recombined into pDEST17 (Invitrogen) according to the manufacturer’s instructions. The His-tagged peptide was purified and injected into rats with complete or incomplete adjuvant (Thermo Scientific/Pierce, Thermo Scientific, Waltham, MA USA).

### Immunostaining

Ovaries were immunostained as described previously [24]. The antibodies used for immunostaining were rat polyclonal anti-Tap (1:1,000), rabbit polyclonal anti-Tej (1:250) [16], guinea pig polyclonal anti-Vas (1:1,000) [16], mouse anti-Aub (1:1,000) [7], mouse anti-Ago3 (1:1,000) [54], rabbit anti-Krimp (1;10,000) [24], guinea pig anti-Mael (1:500) [55], mouse anti-Gurken 1D12 (1:10) (Hybridoma Bank, Iowa City, IA, USA), rabbit anti-Qin (1:1,000) [26], mouse anti-Piwi (1:1) (from Dr Siomi), rabbit anti-Armitage (1:1,000) [56], guinea pig anti-Rhino (1:1,000) [57], mouse anti-Myc (1:1,000) (Sigma, St. Louis, MO, USA), rabbit anti-Ste (1:1,000) [58], rabbit anti-α spectrin (1:2,500) [59] and guinea pig anti-Bam-C (1:200, from Dr McKearin). Secondary antibodies were Alexa Fluor 488-, 555-, 633-conjugated goat anti-rabbit, anti-mouse, anti-rat and anti-guinea pig IgG (1:400) (Molecular Probes, Eugene, Oregon, USA). Images were acquired with a Carl Zeiss Exciter confocal microscope, Oberkochen, Germany and processed in Adobe Photoshop.

### TUNEL assay

The TUNEL assay was performed using an ApopTag® Fluorescein In Situ Apoptosis Detection Kit (Millipore, Billerica, Massachusetts, USA) in accordance with the manufacturer’s protocol.

### Western blot analysis and immunoprecipitation

Ovaries were dissected in Grace’s medium and processed for IP as described previously [16]. For western blotting, one-half ovary equivalent lysate was loaded into each lane of an 8% or 10% SDS-PAGE. The following primary antibodies were used: mouse anti-Aub (1:1,000, from Dr Siomi), mouse anti-Ago3 (1:500, from Dr Siomi), rat anti-Tap (1:1,000, this study), mouse anti-c-Myc 9E10 (1:5000, Sigma), mouse anti-HA (1:5,000, Roche, BASEL, Switzerland), mouse anti-FLAG M2 and its horseradish peroxidase (HRP)-conjugated secondary antibody (1:1,000, Sigma), guinea pig anti-Vas (1:5,000) [16], rabbit anti-Piwi (1:500, Abcam, Cambridge, England, United Kingdom Ab5207), mouse anti-Piwi (1:50, from Dr Siomi), rabbit anti-SpnE (1:500, from Dr Dahua Chen) and mouse anti-α-Tubulin DM1A (1:1,000, Santa Cruz Biotechnology, Santa Cruz Biotechnology, Dallas, Texas, U.S.A.). Immunoreactive bands were visualized using HRP-conjugated goat anti-guinea pig (Dako, Dako North America, Inc. Carpinteria, CA, USA), anti-rabbit, anti-rat or anti-mouse secondary antibodies (Bio-Rad, Hercules, CA, United States of America) at 1:5,000, and developed with the SuperSignal West Pico Chemiluminescent Substrate detection reagent (Thermo Scientific).

### S2 cell experiments

pENTR™/D-TOPO containing Tap, Tej or Ago3 was recombined into either pAFW or pAMW (The Drosophila Gateway Vector Collection, Carnegie Institution for Science Baltimore, Maryland, USA) following manufacturer’s protocol. Vas-V5, FLAG-Tej and Myc-SpnE generation were described previously [16]. Transfections and IPs with S2 cells lysates were performed as described previously [16].

### Real-time RT-PCR

Total RNA was extracted from ovaries or testes with TRIzol (Invitrogen) according to the manufacturer’s protocol. Real-time RT-PCR was performed as described previously [24]. The primer sequences for *HetA*, *TART*, *I-element*, *actin5C, roo* and *ZAM* were described in [24].

### Small RNA sequencing and analysis

RNAs were extracted from hand-dissected ovaries of *tej*^*48–5*^, *tap*^*125*^ and *tej*^*48–****5***^***-****tap*^*125*^, and their corresponding heterozygotes. Small RNAs ranging from 18 to 30 nucleotides were isolated by PAGE fractionation and were used for library generation for deep sequencing. Deep sequencing was performed on HiSeq2000 at Macrogen Inc. (Seoul, Korea). All six libraries were normalized with noncoding RNAs derived from snoRNAs [60], endogenous siRNAs [10] and noncoding RNAs (Additional file 2: Table S1). The libraries were mapped to *Drosophila* genome (Rel 5, excluding Uextra) without any mismatches. To analyze piRNA matching to clusters, only 23- to 29-nucleotide reads that uniquely mapped to the genome were considered (cluster information was taken from Brennecke *et al*. [6]). The piRNAs mapping to the clusters were counted in 10-nucleotide windows for plotting. The libraries were mapped to transposons allowing two mismatches. To analyze ping-pong generated piRNAs, we calculated sense-antisense piRNA pairs having an overlap between 2 and 26 nucleotides using in-house programs. The ping-pong ratios were calculated by dividing the numbers of reads containing a 10-nucleotide overlap with the sum of reads containing any overlap between 2 and 26 nucleotides.

### Availability of supporting data

The small RNA deep-sequencing libraries without 30-nucleotide rRNA reads from the single and double mutants and their corresponding heterozygotes were deposited in the National Center for Biotechnology under the accession number [SRP044384]. The untrimmed raw fastq files are available upon request.

## Additional files

Additional file 1: Figures S1-S6. Supplementary figures. Additional file 2: Table S1. Normalization of small RNA libraries. Additional file 3: Table S2. Cluster mapping small RNAs. Additional file 4: Table S3. Small RNAs mapping to major transposon families. Additional file 5: Table S4. Reduction of piRNAs with 10-nucelotide overlap in *tap*-mutant ovaries.

## Abbreviations

dpe: days post eclosion
GFP: green fluorescent protein
GSC: germline stem cells
HRP: horseradish peroxidase
IgG: immunoglobulin G
IP: immunoprecipitation
kDA: kiloDaltons
piRNA: Piwi-interacting RNA
RT-PCR: reverse transcription polymerase chain reaction
siRNA: small interfering RNA
TE: transposable elements
TUNEL: terminal deoxynucleotidyl transferase-mediated dUTP nick-end labeling
