# Supplementary material for: The Tudor domain protein Tapas, a homolog of the vertebrate Tdrd7, functions in the piRNA pathway to regulate retrotransposons in germline of Drosophila melanogaster
Source: BMC Biol. 2014 Oct 6;12:61. doi: 10.1186/s12915-014-0061-9 (PMC4210518; doi:10.1186/s12915-014-0061-9)
Supplement: Additional file 1: Figures S1-S6. — Supplementary figures. [file 12915_2014_61_MOESM1_ESM.pdf]

Fig. S1

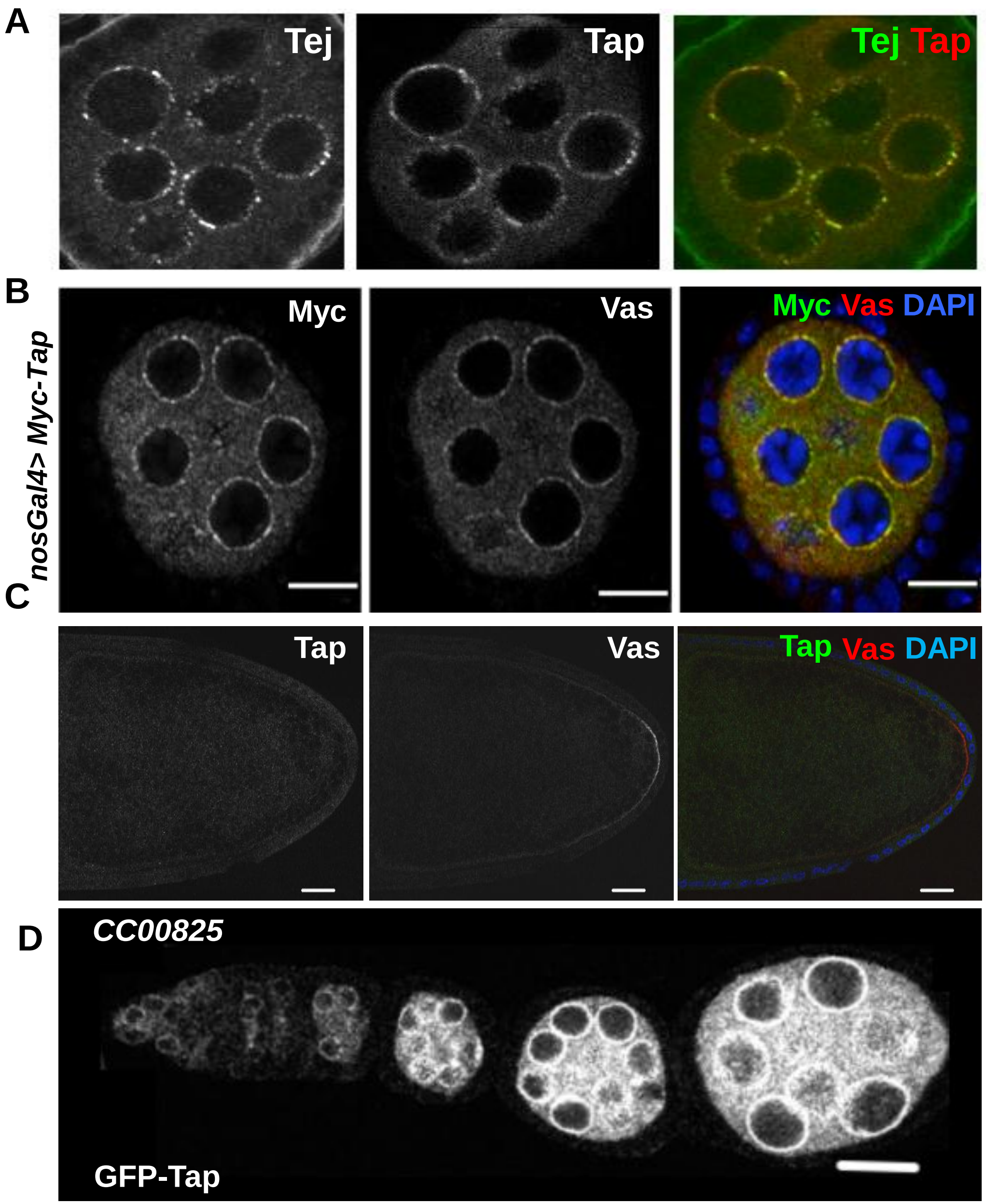

Fig. S1: Tap localizes to nuage but is not a pole plasm component. (A) An egg chamber stained for Tej (green) and Tap (red) showing co-localization. (B) Myc-Tap (green) colocalizes with Vas (red) at perinuclear nuage in germline cells. Scale bar = 5 µm. (C) A wild-type stage 10 egg chamber immunostained for Tap (green) and Vas (red). Unlike Vas, Tap did not localize to the posterior pole. Scale bar = 10 µm. (D) A Tap protein trap line, *CC00825*, expressing GFP-Tap was immunostained for GFP. GFP-Tap localized to the perinuclear nuage. Scale bar = 5 µm.

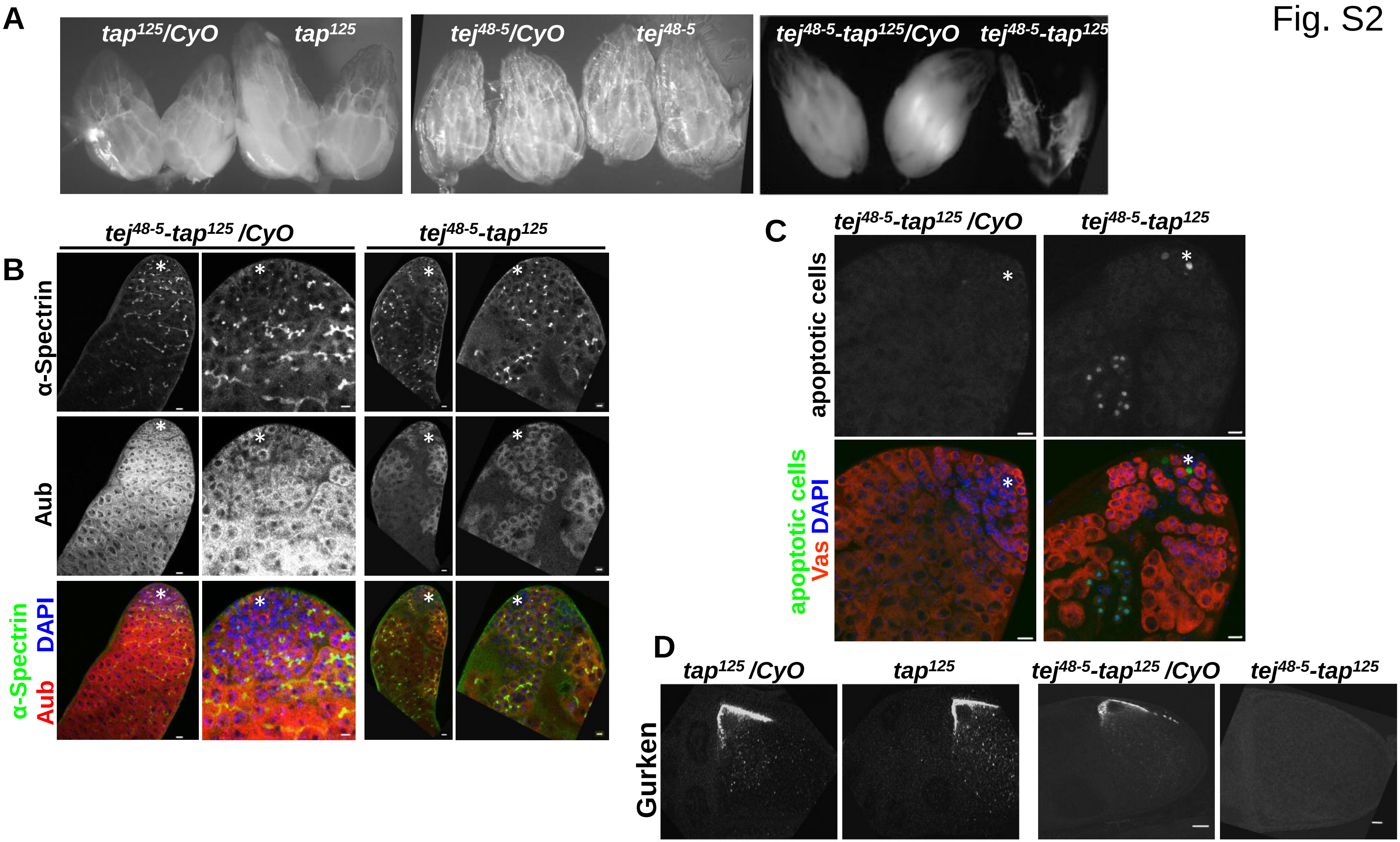

Fig. S2: *tej-tap* loss results in severe defects in germline development. (A) Seven days old ovaries from *tap*^{125}, *tej*$^{48\text{-}5}$ and *tej*$^{48\text{-}5}$-*tap*^{125} , loss of both *tej* and *tap* together leads to degeneration of ovaries. Testes from the *tej*$^{48\text{-}5}$-*tap*^{125} double mutant and the heterozygous control immunostained for Vas showing loss of germline cells. (B) Testes immunostained for α-Spectrin (green), Aub (red), and DAPI (blue). Unlike the heterozygote control, *tej*$^{48\text{-}5}$-*tap*^{125} double-mutant testes contained fewer germline cysts containing fragmented fusomes. Asterisks denote the hub. Scale bar = 20 µm. (C) TUNEL staining (green) of the heterozygous control and the *tej*$^{48\text{-}5}$-*tap*^{125} mutant testes with Vas (red) and DAPI (blue). Scale bar = 10 µm. (D) Immunostaining for dorsal marker Gurken in *tap*^{125} and *tej*$^{48\text{-}5}$-*tap*^{125} mutant and their heterozygote control indicates proper Gurken localization in *tap*^{125} ovaries, while Gurken expression was undetectable at the anterior dorsal side of stage 7 egg chambers in the *tej*$^{48\text{-}5}$-*tap*^{125} double mutants. Scale bar = 5 µm.

Fig S3

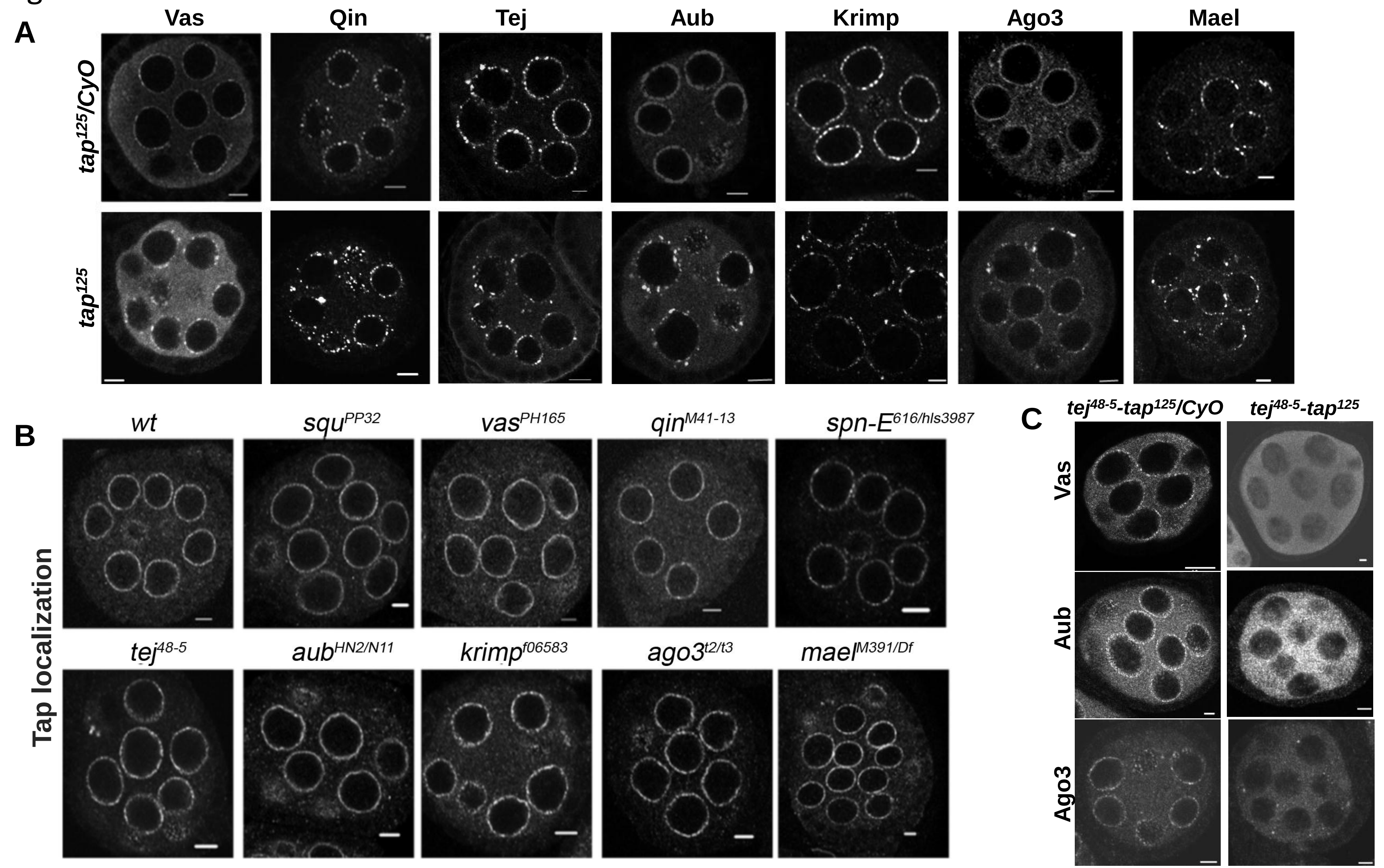

Figure S3: The tap and tej function together for localization of other piRNA pathway components. (A) Immunostaining for nuage components in *tap*^{125} mutants and heterozygous controls. They often form larger foci in *tap* mutant than those controls at perinuclear nuage. (B) Tap in piRNA pathway component mutants remains at characteristic perinuclear region as observed in corresponding heterozygous controls. (C) Aub, Ago3 and Vas are displaced from perinuclear nuage in *tej*$^{48\text{-}5\text{-}}$*tap*^{125} mutant germline cells. Scale bar = 5 μm.

Fig S4

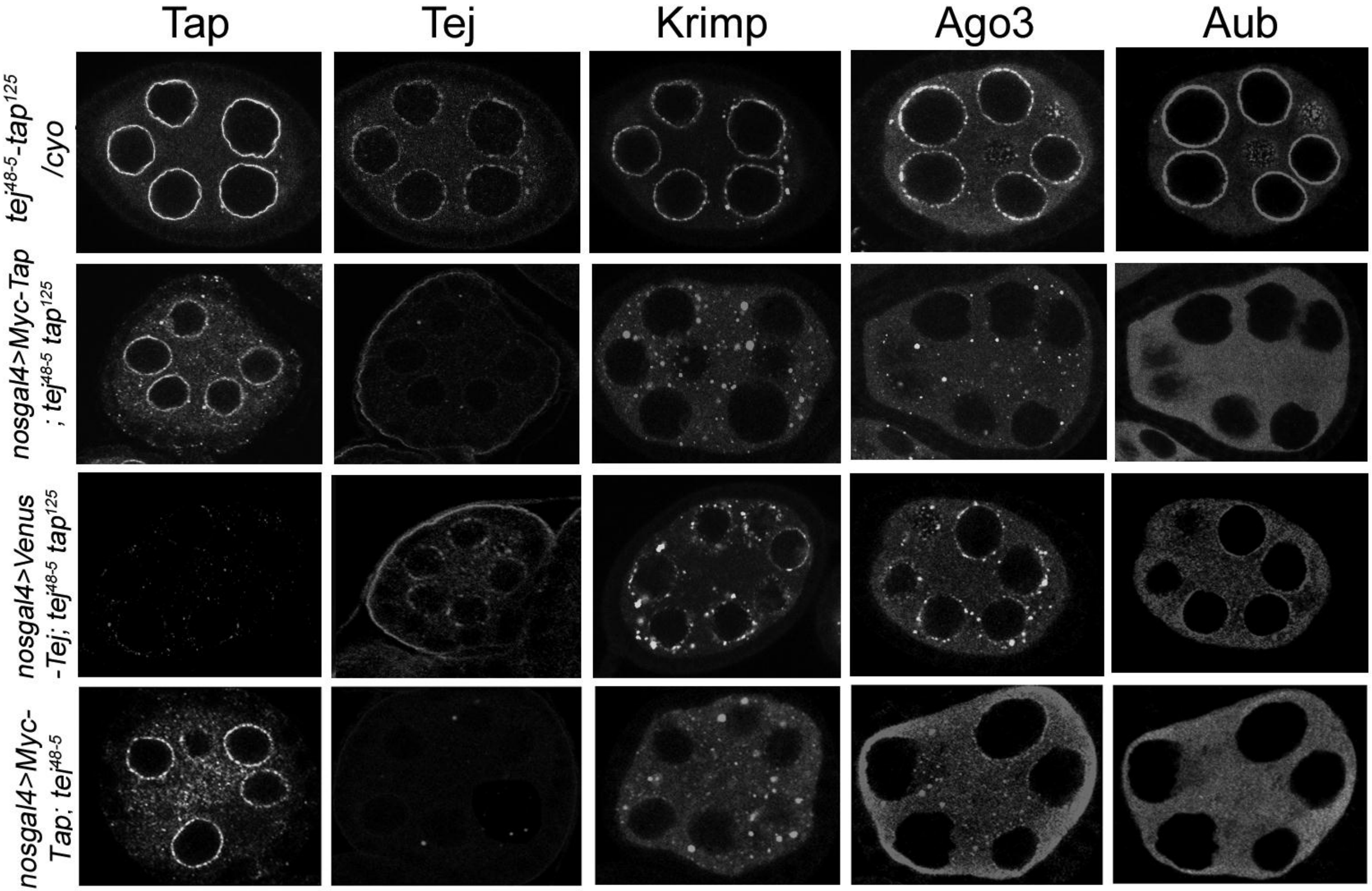

Figure S4: Tej and Tap may function synergistically for localization of piRNA pathway components. Expression of Tap in *tej* mutant female germline or expression Tej and Tap separately in female germline cells of double mutants. Tap overexpression in *tej* or double mutants does not rescue localization of piRNA pathway components to nuage. While Tej overexpression in double mutants brings back piRNA pathway components to nuage though they for larger foci as seen in *tap* mutants.

Fig S5

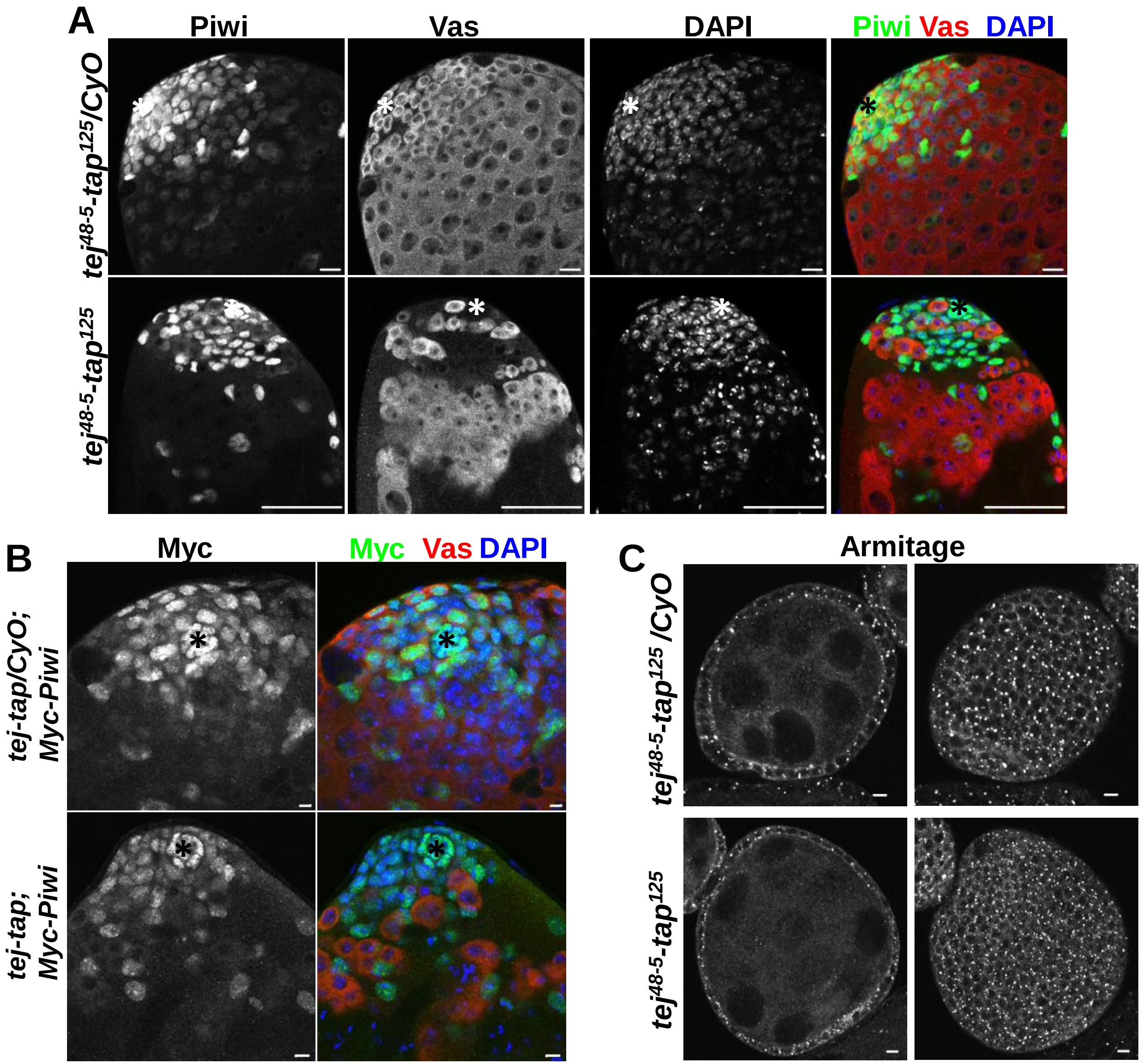

Fig. S5: Tej and Tap loss affects Piwi nuclear localization only in germline cells. (A) Testes immunostained for Piwi (green), Vas (red), and DAPI (blue), shows Piwi was displaced from the nucleus of Vas-positive germline cells but remained unaffected in somatic cell *tej*$^{48\text{-}5}$-*tap*^{125} double mutant, while in both single mutants Piwi localization was comparable to controls. (B) Myc-Piwi expressed from its native promoter remained in the cytoplasm of the *tej*$^{48\text{-}5}$-*tap*^{125} mutants testis. Scale bar =5µm. (C) Egg chambers of the heterozygous control and *tej*$^{48\text{-}5}$-*tap*^{125} stained for Armitage. Focal sections in the middle (left panels) and those at the top (right panels) of each chamber are shown. Scale bar = 5 µm.

Fig S6

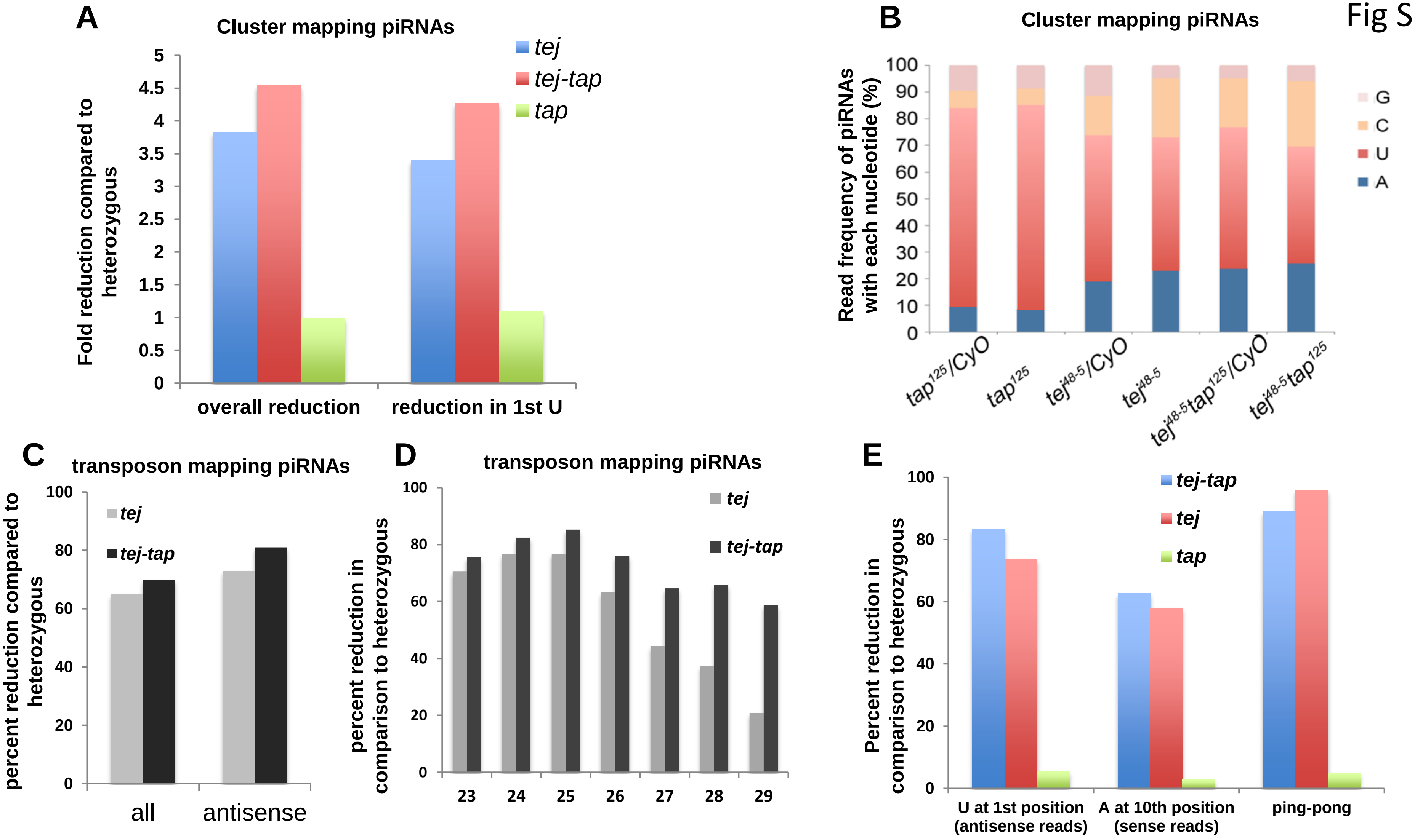

Figure S6: The more severe reduction in piRNAs in double mutants indicate *tej* and *tap* likely function together for the piRNA production. (A) A bar plot shows the double mutant suffer from most severe reduction in piRNAs mapping to clusters. (B) Frequency of nucleotide at 1st position of cluster mapping piRNAs from all three mutants and corresponding heterozygous controls, indicate that the $tej^{48\text{-}5}$-tap^{125} mutants result in reduction of cluster mapping piRNAs with U at 1st position. (C) A bar plot showing percent reduction in transposons-mapping overall, and antisense piRNAs in $tej^{48\text{-}5}$ and $tej^{48\text{-}5}$-tap^{125} mutants compared to their respective heterozygous controls. (D) Reduction in each transposon-mapping piRNA for sizes 23-29 nt in $tej^{48\text{-}5}$ and $tej^{48\text{-}5}$-tap^{125} mutants compared to their respective heterozygous controls. Loss of *tej* and *tap* both causes greater reduction in piRNAs from 26-29 nt in length. (E) Reduction in transposon-mapping anti sense piRNAs with U at 1st position, those sense piRNAs with A at 10th position and those with ping-pong pairs. $tej^{48\text{-}5}$-tap^{125} double mutants shows greater loss in all three piRNAs, indicating that *tej* and *tap* are required together for the primary and secondary processing.
